# PreserFlo^®^ MicroShunt: An Overview of This Minimally Invasive Device for Open-Angle Glaucoma

**DOI:** 10.3390/vision6010012

**Published:** 2022-02-09

**Authors:** Gloria Gambini, Matteo Mario Carlà, Federico Giannuzzi, Tomaso Caporossi, Umberto De Vico, Alfonso Savastano, Antonio Baldascino, Clara Rizzo, Raphael Kilian, Aldo Caporossi, Stanislao Rizzo

**Affiliations:** 1 Ophthalmology Unit, Fondazione Policlinico Universitario A. Gemelli, IRCCS, 00168 Rome, Italy; tomaso.caporossi@gmail.com (T.C.); umbertodevico@gmail.com (U.D.V.); alfonso.savastano@policlinicogemelli.it (A.S.); antonio.baldascino@policlinicogemelli.it (A.B.); aldocaporossi@yahoo.it (A.C.); stanislao.rizzo@gmail.com (S.R.); 2 Ophthalmology Unit, Catholic University “Sacro Cuore”, 00168 Rome, Italy; 3 Ophthalmology, Department of Surgical, Medical and Molecular Pathology and Critical Care Medicine, University of Pisa, 56126 Pisa, Italy; clararizzo2@gmail.com; 4 Ophthalmology Unit, University of Verona, 37134 Verona, Italy; raphaelkilian8@yahoo.it

**Keywords:** glaucoma, micro-invasive glaucoma surgery, MicroShunt, mitomycin C, SIBS polymer, glaucoma drainage devices, InnFocus MicroShunt, PreserFlo, PreserFlo MicroShunt, XEN Gel Stent

## Abstract

For moderate-to-severe glaucoma, trabeculectomy remains the “gold standard” intraocular pressure (IOP)-lowering treatment; nonetheless, this method requires extensive post-operative maintenance. Microinvasive glaucoma surgery (MIGS) treatments are designed to lessen intra- and post-operative care burden while offering an acceptable IOP decrease for individuals with mild to moderate glaucoma. The PreserFlo^®^ MicroShunt (previously InnFocus MicroShunt) is an 8.5 mm glaucoma drainage device manufactured from poly(styrene-block-isobutylene-block-styrene) (SIBS), an extremely biocompatible and bioinert material. The lumen is narrow enough to prevent hypotony, but big enough to avoid being obstructed by sloughed cells or pigment. The device is implanted ab externo, as a stand-alone procedure or in conjunction with cataract surgery, with intraoperative mitomycin C, and a bleb is produced under the conjunctiva and Tenon’s capsule. The MicroShunt was CE-marked in 2012 and designed for primary open-angle glaucoma, the IOP of which remains uncontrolled after maximally tolerated topical treatment. Several clinical trials evaluating the MicroShunt’s long-term safety and effectiveness have been conducted, highlighting the effectiveness of the device over time, along with a tolerable safety profile. The present review aims to gather evidence of PreserFlo’s effectiveness and safety results almost 10 years after its introduction, and furthermore, to compare it with other MIGS and with the gold-standard trabeculectomy for glaucoma management.

## 1. Introduction

Glaucoma is a neurodegenerative disorder marked by the death of retinal ganglion cells and cupping of the optic nerve head, both of which cause vision field loss [1,2]. The most prevalent kind of glaucoma is primary open-angle glaucoma (POAG). The only well-established modifiable risk factor is intraocular pressure (IOP), and medicinal therapy is often used to reduce IOP in the treatment of glaucoma. Patient adherence to medicine, on the other hand, might be poor. In order to improve long-term IOP reduction, laser and incisional surgical techniques, including trabeculectomy and tube shunt surgery, have been introduced [3,4,5,6]. Both trabeculectomy and tube shunt surgery are invasive operations that need a significant amount of postoperative care [3,4,5].

Micro-invasive glaucoma surgery (MIGS), known also as minimally invasive glaucoma surgery (MIGS), defines a growing number of surgical techniques for glaucoma [7]. MIGS operations attempt to minimize intraoperative and postoperative management, as well as provide a less intrusive method of lowering IOP than standard glaucoma surgery, with the objective of minimizing reliance on topical drugs [8]. MIGS are able to reduce IOP exploiting different anatomical pathways: (1) boosting trabecular outflow by bypassing the trabecular meshwork and directly involving Schlemm’s canal, (2) lowering ciliary body aqueous production; (3) increasing uveo-scleral outflow through suprachoroidal routes, or (4) creating a link between the anterior chamber and the subconjunctival space to improve aqueous humor drainage and forming a bleb. [7,8] Although MIGS procedures have been claimed to have a better safety profile than traditional surgery, the variability among devices, with differences in the outflow pathway, ab interno versus ab externo approach, and whether a bleb is created, reflects a diverse target glaucomatous population, efficacy, and device- or procedure-related adverse events (AEs) [7,8,9].

On one side, most MIGS treatments without bleb formation have only been linked with minor decreases in IOP and are therefore focused on patients with mild-to-moderate glaucoma, thus indicating an unmet need for minimally invasive treatment of moderate-to-severe and refractory glaucoma [8,10]. MIGS devices that result in the formation of a bleb, on the other hand, have been linked to significant IOP reductions. Despite this, postsurgical management of the bleb, such as early needling with or without the concomitant injection of mitomycin C (MMC) to mitigate fibrosis, has a crucial role in the success rate of these devices [11,12]. Nevertheless, concerning these new devices, long-term IOP data are still lacking [10].

The discovery of a novel synthetic thermoplastic elastomeric biomaterial (poly(styrene-block-isobutylene-block-styrene); SIBS) lead to the introduction of a SIBS-based glaucoma device named PreserFlo MicroShunt (MicroShunt, formerly known as the InnFocus MicroShunt) [13,14]. This product received Conformité Européenne marking in 2012, Health Canada and Therapeutic Goods Administration of Australia approval in 2021, and a US Investigational Device Exemption (IDE) to initiate a Phase 3 clinical study was granted by the US Food and Drug Administration (FDA) in May 2013. At the moment, the PreserFlo is still an investigational device not yet approved by the Food and Drug Administration.

Structurally speaking, SIBS has the advantage to resist biodegradation in the body, along with the necessity for a safe and efficient means of treating glaucoma. The PreserFlo MicroShunt is an 8.5-mm-long glaucoma filtration surgical device with a 350-μm outer diameter and a 70-μm lumen that is implanted through an ab externo technique. The device’s proximal tip rests in the anterior chamber, parallel to the iris, while the distal tip sits under the conjunctiva and Tenon’s capsule, about 6 mm beyond the limbus, enabling aqueous humor to pass through the lumen to produce a posterior bleb after implantation [14,15].

## 2. History of PreserFlo MicroShunt

An iterative 20-year study was needed to give birth to SIBS material and, later, to the PreserFlo MicroShunt [16]. Pinchuk was the first to introduce SIBS at the University of Miami’s Miller School of Medicine, Bascom Palmer Eye Institute, Optical Biophysics Center, sometime around 2003 [13,14].

**Figure 1:**
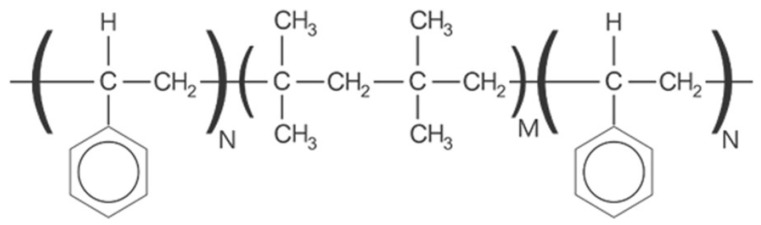
Simplified chemical structure of SIBS. M = number of isobutylene units; N = number of styrene units; SIBS = poly(styrene-block-isobutylene-block-styrene).

In those years Parel et al. implanted 3 mm diameter, 1 mm-thick SIBS disks in the corneal stroma, as well as beneath the conjunctiva and Tenon’s capsule of white rabbits’ eyes. In the control group, silicone rubber (polydimethylsiloxane) disks were implanted alongside the SIBS disks. The biocompatility of this material was evaluated at the 2-month control, and no myofibroblasts or angiogenesis in the region of the SIBS disks, as well as no integral capsules around the disks was described [17]. Few years after, Acosta and colleagues presented similar findings, proving in conclusion that SIBS was completely harmless to the eyes [18] (Figure 1).

These researchers claimed that SIBS material could be used for a glaucoma drainage device, but optimal constructive features were necessary. To avoid clogging, the lumen needed to be greater than the diameter of a sloughed endothelial cell, which is about 40–50 um, while remaining small enough to avoid eye trauma and hypotony. The Hagen–Poiseuille equation was used to estimate the lumen size, and Arrieta et al., in a series of rabbit eye implants, demonstrated that a lumen diameter of 70 μm would meet these parameters [19,20]. Moreover, Pinchuk et al. highlighted that device drainage to a flap beneath the conjunctiva and Tenon’s capsule, comparable to the trabeculectomy bleb, was the best approach [14,21]. These good preclinical results in ophthalmology were consistent with SIBS experience, previously adopted in cardiology. In fact, the SIBS coated TAXUS^®^ (Boston Scientific Corporation, Natick, MA, USA) was a cardiac stent that minimizes restenosis by releasing the antiproliferative medication paclitaxel in the coronary artery [22]. TAXUS^®^ has been implanted in over a million patients throughout the globe and has a proven safety record, showing negligible biodegradation and low inflammation in vitro and in vivo experiments, demonstrating SIBS’ flexibility as a biocompatible polymer [14,22,23].

Before arriving at the present MicroShunt concept, three significant variations in the design were studied, with the first two to be investigated in acute and chronic rabbit eye biocompatibility studies [13,14]. The first approach was the Miami InnFocus Drainage Implant (MIDI)-Tube, an 11 mm SIBS tube with a 1 mm SIBS tab, firstly evaluated in two studies at the Bascom Palmer Eye Institute Ophthalmic Biophysics Center (OBC) (Miami, FL, USA), and then confirmed in a good laboratory practice (GLP) study at the North American Science Associates contract facility (Northwood, OH, USA) [14,21]. The second variation was named MIDI-Ray (a 350 µm diameter, 100 µm lumen, SIBS tube with a 7 mm diameter SIBS plate) and then studied in chronic, non-GLP animal research at the Bascom Palmer Eye Institute OBC [14].

These SIBS-based devices were subsequently put through clinical testing after receiving good findings from biocompatibility tests. Four human pilot feasibility studies (Bordeaux I and II, and Dominican Republic I and II) were needed in the successive 4 years to determine the optimum design, implantation procedures, and whether mitomycin C (MMC) use was necessary or not [14]. The MicroShunt technique along with MMC injection (0.4 mg/mL for 3 min using sponges), was claimed to be successful in 95% of cases in the Dominican Republic II research, and was chosen for further clinical testing [14,15]. The final design, known as the InnFocus MicroShunt, was made up of an 8.5 mm-long SIBS tube with a 350 um outer diameter and a 70 μm lumen diameter. 

## 3. Surgical Technique

The MicroShunt is implanted through an ab externo route, and the surgical process is minimally invasive when compared with trabeculectomy [13,24]. Aqueous humor drained from the anterior chamber is directed via the MicroShunt to a bleb produced under the conjunctiva and Tenon’s capsule. The reabsorption of the subconjunctival fluid collected inside the bleb follows different pathways: (1) the episcleral venous system [13]; (2) the tear film through microcysts, which are naturally occurring conjunctival channels [13,25]; and (3) via orbital lymphatics [26,27]. The MicroShunt thus overcomes the significant resistance of the trabecular meshwork, Schlemm’s canal, collecting channels, and scleral venous plexus by draining aqueous humor down this pathway [13,24].

At the nasal or temporal superior quadrants, a fornix-based subconjunctival and sub-flap Tenon’s is dissected throughout a circle of 90 to 120 degrees, to at least 8 to 10 mm posterior to the limbus [24]. After this, mitomycin C (MMC)-soaked sponges are placed in the flap for 2 to 3 min of exposure. In minimally invasive devices, such as the MicroShunt, intraoperative application of MMC has been proven to lower the chance of surgical failure and raise the surgical success rate [13]. Various concentrations and application durations of MMC during MicroShunt implantation have been described, mostly focusing on concentrations of 0.2–0.4 mg/mL and application periods of 2–3 min [13,15,28].

**Figure 2:**
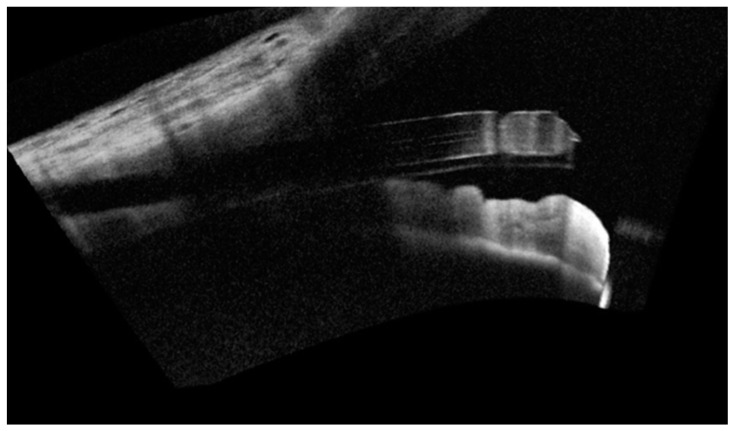
Anterior segment—optical coherence tomography (AS-OCT) showing successfully implanted PreserFlo MicroShunt, piercing the trabecular meshwork and positioned bevel up in the anterior chamber.

Successively, after an abundant rinsing with saline solution, a marker is used to indicate a position 3 mm from the middle border of the surgical limbus in the blue-gray zone. A 1 mm knife is used to incise a shallow triangular pocket in the sclera (big enough to host the MicroShunt fins) at the distally marked position. The apex of the scleral pocket is then punctured with a needle in order to create a transscleral tunnel into the anterior chamber. The MicroShunt is finally inserted into the transscleral tube with forceps, bevel up and fins flat [24] (Figure 2).

After that, the fins are squeezed into the scleral pocket. Prior to Tenon’s capsule and conjunctiva closure, it is critical to evaluate flow via the MicroShunt. Successful flow is visually established by first seeing a percolation of aqueous humor from the device’s distal end, just after air purge from the lumen. Flow may seem to decrease as the volume of the drop grows; nevertheless, volume increases to the third power of flow, making flow difficult to measure when the drop is too great. In that case, it may be useful to wipe the drop away with a sponge now and then and imagine a little drip to ensure flow [24]. The target IOP in that moment should reach about 6 mmHg or less at equilibrium flow, which may be achieved by depressing the central cornea using a 30 g cannula or using a Schiötz tonometer. 

**Figure 3:**
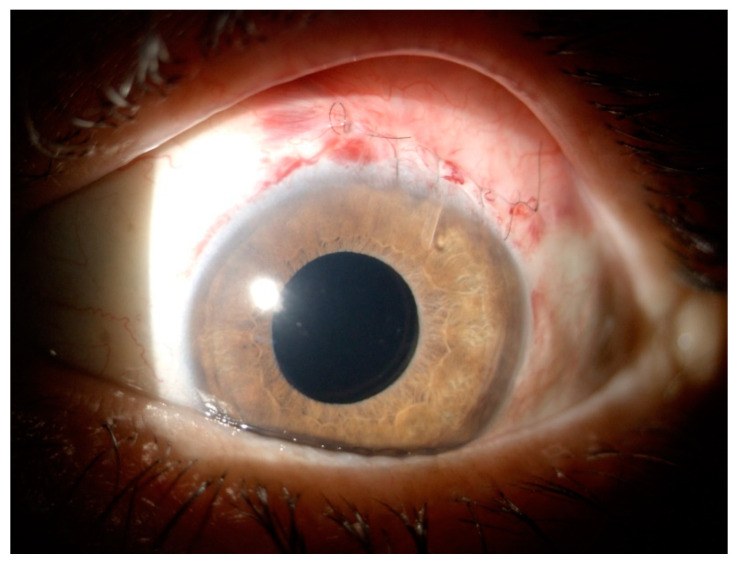
PreserFlo MicroShunt the day after surgical implant. Pretty visible tube in the anterior chamber, sutures over the conjunctiva and the forming filtering bleb.

Different approaches can be applied, whether flow is seen through the lumen or not. Firstly, it is crucial to ensure that the MicroShunt’s entry is clean of debris and not trapped in the iris or cornea. After this, a BSS injection in the anterior chamber may increase IOP to assure MicroShunt percolation functionality. If still not working, a 30 g cannula may be used to inject BSS into the MicroShunt’s lumen to release air and prime the device. It is also important to check whether the fins are properly installed, because fluid flow around the MicroShunt may indicate that the path of minor fluid resistance may be around the device rather than into the lumen. Device withdrawal, if the fins are wedged too tightly, or new tunnel creation may be needed for troubleshooting when the aforementioned precautions are shown to be ineffective [24]. Following flow confirmation, the MicroShunt’s distal end is tucked under Tenon’s capsule and conjunctiva. After ensuring that the device is straight and free of tissue, sutures are necessary to reattach Tenon’s capsule and conjunctiva over the device and to the limbus [13,15] (Figure 3).

PreserFlo MicroShunt may be implanted in conjunction with cataract surgery or as a stand-alone treatment. Everywhere, its minimally invasive approach highlights no need for intraoperative gonioscopy, sclerotomy, or iridectomy [29,30,31].

## 4. PreserFlo MicroShunt Results

The PreserFlo MicroShunt is a device whose utility has been demonstrated over time. The first notable clinical data about the surgical success of this technique were published in 2014, when Riss et al. presented the results of a retrospective two-center, two-surgeon analysis with a one-year follow-up after InnFocus MicroShunt implantation [32]. A total of 87 eyes with primary OAG were selected, with 21 of them receiving cataract surgery as well. The primary goal of this study was to see whether MMC concentration and placement location affected the procedure’s efficacy, so 31 eyes (35.6%) received 0.2 mg/mL MMC near the limbus, 23 (26.4%) received 0.4 mg/mL MMC near the limbus, and 33 (37.9%) received 0.4 mg/mL MMC deep in the conjunctival pocket, all for 2–3 min [32]. Overall, the mean preoperative IOP was 25.9 mmHg with an average of 2.6 topical medication, which dropped to 13.5 mmHg on 0.56 medications. The authors did not show any statistical data, despite suggesting that greater MMC dose and positioning near the limbus were related to the largest IOP decrease. Furthermore, no baseline data were compared across the groups, nor was there a breakdown of which eyes received combined cataract surgery [32].

In order to describe the Innfocus MicroShunt as a viable alternative to trabeculectomy, particularly in terms of efficacy and safety in decreasing IOP, Pinchuk et al. collected the 23 eyes receiving 0.4 mg/mL MMC near the limbus from the Riss research, and followed them prospectively with postoperative data at two years of follow-up [13,32]. This cohort was composed of primary OAG patients from the Dominican Republic, of which 14 eyes (60.9%) received standalone InnFocus MicroShunt and 9 (39.1%) were combined with cataract surgery. The “qualified success” rate, as defined by IOP < 18 mm Hg and a 20% drop in pressure from baseline, was 100% at both follow-ups. However, no visual acuity variations were observed in any of the patients who underwent glaucoma surgery alone over this period studied. Following the implantation of a MicroShunt in conjunction with cataract surgery, three patients gained two or more lines after one year, and four patients gained two or more lines after two years [13]. IOP < 5 mm Hg after day 1 was the most common post-operative adverse event, regarding 13.0% of all cases. Three of the 23 patients had shallow anterior chambers; however, none of them required anterior chamber reformation or choroidal effusion drainage. Two patients (8.7%) from the combined group had choroidal detachment, which resolved spontaneously. No long-term serious adverse effects were reported [13].

Moreover, on the same 23-eyes cohort, Batlle and colleagues conducted a three-year follow-up. The criteria for “complete success” (IOP ≤ 21 mmHg, IOP decrease ≥ 20% from baseline, no reoperation for glaucoma or supplemental medication) and “qualified success” were adopted from the tube versus trabeculectomy (TVT) study [3]. At three years of follow-up, the qualified success rate (IOP ≤ 14 mm Hg and IOP reduction of at least 20%) was 95 percent. An average baseline IOP 23.8 ± 5.3 was reported, with mean IOPs reaching 10.7 ± 2.8, 11.9 ± 3.7, and 10.7 ± 3.5 mm Hg at the three annual follow-ups. In addition, the mean number of glaucoma medications per patient was reduced from 2.4 to 0.7 at the last follow up. No other long-term adverse effects were seen [15]. 

Batlle again reported the results of the same cohort at the 4- and 5-year follow-up, in a more recent publication [33]. The study protocol was indeed changed to make it a five-year study, with IOP decreasing from 23.8 mmHg on 2.4 medicines to 12.8 mmHg on 0.3 medicines at year 4 and 12.4 mmHg on 0.4 medicines at year 5, demonstrating a mean drop from the baseline of 45.7 and 46.7%, respectively. Following MicroShunt implantation, >80% of patients had overall success with an IOP of between 6 and 21 mmHg at years 4 and 5 [33]. Among all patients, 13 suffered from 31 non-serious adverse events (AEs). In two patients, four significant procedure- or device-related adverse events were reported, all resolving in a maximum of 20 days. The most serious adverse events were posterior capsule opacification (*n* = 2), posterior synechiae (*n* = 1), and pupillary capture (*n* = 1). Bleb failures necessitated reoperation in two patients (8.7%); in one of these patients, a second MicroShunt was implanted; in the other, the MicroShunt was replaced with a 45 μm XEN Gel Stent (Allergan, Dublin, Ireland). In two individuals, bleb needling was necessary. No complaints of device movement or over-conjunctival exposure were recorded [33].

Beckers et al., in 2017, published a brief retrospective abstract that summarized 12-month data from a three-site (France, Dominican Republic, and Netherlands) study, in which 91 consecutive primary OAG eyes undergoing standalone InnFocus (38 phakic and 35 pseudo-phakic eyes) or combined InnFocus and cataract surgery (18 eyes), were gathered [34]. Preoperative IOP was 24.3 mmHg on 2.4 drugs, dropping to 13.3 mmHg on 0.4 medications at the 12-month follow-up. Interestingly, 83% of patients were post-operatively free from glaucoma medications. Ten patients experienced a transient hypotony ≥ 6 mmHg, as well as some minor hyphema, which self-resolved after three months. Finally, the authors stated that “many cases” necessitated needling or bleb modification to further reduce IOP [34].

Schlenker et al. led a retrospective interventional case study on 164 eyes of 132 patients for one year following PreserFlo MicroShunt implantation [35]. Although reporting a 76.9% rate of complete success and 92.5% of qualified success, the studied cohort was quite inhomogeneous. Only 53% of the people in the group were Caucasian, and only 67% of them had POAG. Needling (8.5%) was the most prevalent intervention within a year of follow-up, followed by AC reformation (3.0%) [35].

Recently, Martinez-de-la-Casa et al., in a retrospective analysis, compared OAG individuals who underwent standalone PreserFlo implantantion (35 eyes) vs. a combination of phacoemultification + PreserFlo implantation (23 eyes) [29]. Overall, mean IOP was shown to reduce from 21.5 ± 3.3 mmHg at baseline to 10.4 ± 3.1 mmHg; 11.6 ± 2.5 mmHg; 13.3 ± 3.3 mmHg; 14.3 ± 3.0 mmHg, and 14.6 ± 3.5 mmHg at 1-week, 1-month, 3-month, 6-month, and 12-month follow-up, respectively. Similarly, the average number of ocular hypotensive medicines was lowered from 2.3 at baseline to 0.2 the last follow-up. Additionally, this research highlighted no differences between PreserFlo MicroShunt alone or in combination with cataract surgery in either IOP lowering or a reduction in the number of hypotensive medications. In the two sub-groups, the complete success rate was 68.6% (24/35 eyes) and 52.2 percent (12/23 eyes), respectively [29].

A novel study to assess the safety and effectiveness of the PreserFlo MicroShunt was led by Beckers [31]. This prospective, single-arm, multicenter clinical trial (Identifier: NCT02177123) regarded six European sites with a 2 years follow up. In the overall population of 81 eyes, mean IOP ± SD was 21.7 ± 3.4 mmHg at baseline, inferior when compared with Batlle’s Dominican Republic cohort [33], and decreased to 14.5 ± 4.6 mmHg at year 1 and 14.1 ± 3.2 mmHg at year 2. Overall success (defined as the absence of two consecutive pressure failures; outside target range or a ≤20% reduction from baseline, with and without supplemental glaucoma medication use) was 74.1% at 1 year. Almost 80% of these patients achieved complete success (intending supplemental glaucoma medications were not required to maintain controlled levels of IOP) [31]. Similar results were reported at the second year follow-up. At the end of the study period, 73.8% of patients were medication-free (regardless of IOP level), with an average 0.5-per-patient medications overall [31]. Postoperative IOP was comparable in the 0.2 mg/mL and 0.4 mg/mL subgroups during the follow-up period, in a post hoc analysis. However, after month 6, there was a tendency toward larger IOP reduction with 0.4 mg/mL MMC compared with the 0.2 mg/mL MMC group. In addition, 90.3% of patients in the 0.4 mg/mL MMC group were medication free at year 2 compared with just half of the patients in the 0.2 mg/mL group, suggesting that the higher MMC concentration might be more appropriate in the eyes of patients with more severe disease [31]. Nonserious AEs linked to the device or procedure were recorded in 11 (34.4%) and 32 (74.4%) patients in the 0.2 mg/mL and 0.4 mg/mL MMC categories, respectively. The majority of nonserious AEs in the 0.4 mg/mL MMC group occurred before month 1, compared with 16% in the 0.2 mg/mL MMC subgroup, and included transient hypotony (16.3%), keratitis 11.6%) and leakage of the wound site based on the Seidel test (7.0%). A low rate of postoperative interventions was observed after MicroShunt implantation. Of the eight patients requiring bleb revisions, two implanted a new glaucoma surgical implant, one underwent trabeculectomy, one case received a second MicroShunt implant, and the last two underwent a flap resuture and a sclerectomy for increased IOP [31].

### PreserFlo MicroShunt in Pseudoexfoliative Glaucoma

In a recent article, Fea et al. conducted a retrospective study on 104 eyes with both POAG and pseudoexfoliative glaucoma (PXG) to evaluate the effectiveness and safety of the PreserFlo MicroShunt implant, excluding any other form of glaucoma [36]. A total of 81 eyes (77.9%) of patients were diagnosed with POAG and 23 eyes (22.1%) were diagnosed with PXG. Mean IOP decreased considerably both in eyes with POAG (25.0 mmHg at baseline vs.14.3 mmHg at month 12), and in eyes with PXG (25.0 mmHg at baseline vs. 13.5 mmHg at month 12. Furthermore, when comparing the mean IOP-lowering impact and the post-operative AEs between POAG and PXG eyes at month 12, no significant differences were found among the two groups, with no long-term sight-threatening adverse events overall [36]. At last, although evidence showed that individuals with PXG suffered from blood–aqueous barrier disruption following intraocular surgery [37,38], which may have increased the risk of early postoperative problems, in this research topical steroids treatment was not shown to be more extended or intense in the PXG subgroup. These results indicate that the MicroShunt might result in a limited inflammatory response and could offer a good safety profile in other types of open-angle glaucoma too [36].

An overview of the PreserFlo MicroShunt results among different researches is visible in Table 1.

## 5. PreserFlo MicroShunt vs. Trabeculectomy

The differences between individuals treated with a tube shunt and those treated with trabeculectomy were highlighted in the tube versus trabeculectomy (TVT) study, a multicenter randomized clinical trial comparing the safety and efficacy of these two approaches with 1-, 3- and 5-year follow-ups in several studies [3,4,5,39,40]. At one year, both surgical treatments reduced IOP similarly, although trabeculectomy required less additional medicinal therapy [3,41]. The trabeculectomy group showed lower IOP results at three years, with no significant difference in surgical failure rates, [40] while at the five years follow up, tube shunt surgery showed a better success rate. Moreover, in the trabeculectomy group, further glaucoma surgery was required more often than tube shunt implantation [4,5]. Those results highlighted the crucial role of tube shunt surgery, suggesting it could be used earlier in the natural history of glaucoma management, with the possibility to offer an alternative to the classical trabeculectomy with comparable effectiveness.

Since the introduction of MIGS, different reviews have focused on the effectiveness of these new devices and analyzed the transition from trabeculectomy to this minimally invasive surgery [42]. In this panorama, the PreserFlo MicroShunt efficacy and safety have recently been compared with trabeculectomy as a primary treatment for POAG. Pillunat’s research yielded the initial results in glaucomatous patients who had undergone PreserFlo implantation alone vs. MMC-augmented trabeculectomy [43]. A total of 52 eyes were studied, with 26 in each group and comparable pre-operative IOPs among the two groups. The MicroShunt group had a median IOP of 10.8 mmHg at 6 months, while the trabeculectomy group had a median IOP of 10.3 mmHg. There were no statistically significant differences in mean diurnal IOP reduction, median diurnal IOP fluctuation and peak diurnal IOP between groups. The trabeculectomy group had a statistically significant greater rate of post-operative interventions than the MicroShunt group, which might balance the higher costs of PreserFlo at time of surgery. None of the patients had any serious side effects [43].

Baker et al. found slightly different results in their novel 2-year randomized, multicenter study, which had as a major endpoint to evaluate a reduction of at least 20% in mean diurnal IOP from baseline [44]. With 395 patients undergoing PreserFlo MicroShunt + MMC vs. 132 patients receiving trabeculectomy + mitomycin, the success rate in the MicroShunt group appeared lower than in the trabeculectomy group (53.9 vs. 72.7%) at the 1-year follow-up. In particular, average IOP reduced from 21.1 mmHg at baseline on 3.1 medications, to 14.3 mmHg on 0.6 medications in the MicroShunt group. In the trabeculectomy group, lower IOPs were reported at the end of the study period (11.1 mmHg) with lower medications (0.3 on average) [45]. On the other side, postoperative AEs, such as transient hypotony (decrease < 6 mmHg) or hypotony requiring intervention, were documented to be significantly lower in the PreserFlo group (28.9% and 2.0%) versus trabeculectomy (49.6% of transient hypotony and 7.6% of patients requiring additional hypotony interventions). When compared with trabeculectomy, MicroShunt implantation needed fewer postoperative procedures, resulting in fewer postoperative visits. Only 6% of patients in the MicroShunt group needed surgical procedures after the first month, compared with patients in the trabeculectomy group, nearly half of whom needed a laser suture lysis during the postoperative period (49%) [44].

The research group led by Quaranta recently highlighted interesting results in the evaluation of the efficacy of PreserFlo MicroShunt in POAG eyes after a single failed trabeculectomy, performed at least 6 months previously [45]. Out of the 31 eyes included, 67.74% of the eyes reached a final IOP ≤ 14 mmHg with an IOP reduction of at least 6 mmHg, defined as complete success. Indeed, mean IOP reduction was 47.93% from baseline to the 1-year follow-up, and the mean number of topical drugs decreased from 3.29 to 0.46. Furthermore, only a few minor complications occurred throughout the postoperative phase, with transient hypotony and choroidal effusion being the most common of those, but generally self-limiting or requiring only medical management [45].

## 6. PreserFlo MicroShunt vs. Other MIGS

In recent years, the success of MIGS has been evident and different studies have been carried out comparing classical trabeculectomy vs. MIGS, but also different MIGS among themselves [46,47]. Unfortunately, few direct comparisons between the PreserFlo MicroShunt and other MIGS have been published. For example, superiority studies between the Hydrus and the PreserFlo, or between the i-Stent and the PreserFlo, are still lacking.

We need to wait until 2021 for the first studies comparing PreserFlo and other MIGS. Scheres et al. indeed evaluated the long-term efficacy and safety of two minimally invasive implants sharing the subconjunctival drainage approach: the XEN45 Gel Stent (Xen) implant and the PreserFlo MicroShunt [48]. The Xen Gel Stent is generally implanted through an ab interno ‘closed conjunctiva’ method and drains aqueous fluid to the subconjunctival region without the use of an extraocular reservoir. The Xen Gel Stent is preloaded in an inserter with a 27 G sharp beveled needle tip [49,50]. The inserter is introduced via a 1.2 mm clear corneal incision in the inferotemporal area, guided across the anterior chamber to puncture the trabecular meshwork, and into the sclera around 2 mm from the limbus. The stent is then deployed into the subconjunctival area and the needle is retracted into the hub using a sliding mechanism on the inserter. In order to reduce the fibrotic response, mitomycin C (MMC) is generally infused subconjunctivally using a tiny (27 or 30 G) sharp needle just before implant injection [51].

In their study, Scheres et al. retrospectively examined the efficacy of PreserFlo and Xen in primary open-angle glaucoma patients. Both methods were effective in lowering IOP and minimizing the usage of antiglaucoma drugs. Specifically, the 82 implants considered were equally divided among the two devices [48]. In 15 (37%) instances, the Xen implant was used in conjunction with cataract excision, compared with 1 (2%) case in the MicroShunt group. The Xen group’s mean IOP reduced from 19.2 mmHg at baseline to 13.3 mmHg at 12 months, and 13.8 mmHg at 24 months. On the other side, in patients who received a MicroShunt, the average IOP reduced from 20.1 mmHg at baseline to 12.1 mmHg (40 percent decrease) at 12 months, and the IOP value was maintained at 12.1 mmHg at 24 months. The mean number of IOP-lowering drugs decreased from 2.5 ± 1.4 and 2.3 ± 1.5 at baseline, to 0.9 ± 1.2 and 0.7 ± 1.1 at 24 months of follow-up in the Xen Gel and MicroShunt subgroups, respectively [48]. Both groups had similar rates of hypotony and early self-limiting hyphema. However, while the number of bleb needlings and further glaucoma filtration surgery rates was comparable among the two groups, the MicroShunt group had a lower rate of post-operative MicroPulse transscleral cyclophotocoagulation. Moreover, curling of the stent was reported in six (15%) instances in the Xen group, moving the device towards the anterior chamber and necessitating adjustment in the operating room. Diversely, the PreserFlo group did not highlight any device exposure or migration. Despite the fact that both implants have a similar drainage system, there are a few differences between them [48]. Those variations between the two implants may derive from the different structural materials and distinct designs, which might affect biocompatibility, foreign body response, and migration after implantation. Furthermore, the two different surgical approaches (ab externo vs. ab interno) and different MMC administration time and position may produce variable post-operative settings [48].

Wagner et al. recently conducted another comparison, which included Xen Gel Stent, PreserFlo MicroShunt and trabeculectomy with MMC in three subgroups of 35 eyes per group [52]. Complete success was 73.5 percent in the trabeculectomy group, 51.4 percent in the XEN group, and 74.2 percent in the PreserFlo group at 6-month follow-up. Numerically speaking, the IOP decrease was of 12.1 mmHg from baseline in the trabeculectomy group, significantly higher than in the other two subgroups (5.8 mmHg higher than the XEN group and 4.8 mmHg higher than the PreserFlo group). In conclusion, all three methods resulted in a sufficiently low post-operative intraocular pressure, showing comparable surgical success after six months, and hence may be evaluated separately for glaucoma therapy [52].

## 7. Adverse Effects of PreserFlo MicroShunt

The PreserFlo MicroShunt was found in previous tests to be extremely successful in lowering IOP, while also having a positive safety profile [15,32,35]. Similarly to other MIGS devices, united by subconjunctival filtration, bleb-related problems are the first to be dealt with. Bleb fibrosis and encapsulation remain the most common causes of surgical failure, despite the widespread use of antimetabolites. A varying percentage of these approaches requires post-operative needle revision (NR), which is a surgical procedure that involves lysing the fibrous adhesion bands between the conjunctiva and the episcleral surface to restore bleb functioning [53]. Although very successful, with a success rate of 48% to 80%, NR may cause a number of side effects, including leakage, filtration failure, and endophthalmitis [54,55]. However, for the best IOP control, careful postoperative bleb treatment is required even for minimally invasive glaucoma surgery.

The early reported adverse effects of PreserFlo included transient hypotony (IOP less than 5 mmHg), anterior chamber shallowing and choroidal effusion, all of which resolved without intervention. Implant complications were usually temporary and self-resolving or could be managed with medical attention [31,33,51].

The PreserFlo implant was associated with a lower incidence of hypotony when compared with traditional glaucoma surgery, but also with other MIGS approaches such as XEN. The incidence of chronic hypotony following trabeculectomy was 23% after three years in the tube versus trabeculectomy study, rising to 31% after five years [5,39]. On the other side, the occurrences of hypotony with the PreserFlo and the 45 μm XEN were equal, as reported by Scheres et al. [48]. Although the risk of hypotony was greater (39%) with PreserFlo than with XEN 45 (24%) in the first week following surgery, the requirement for anterior chamber reconstruction was lower (2%) with PreserFlo when compared with XEN 45 (5%), with the same 2% rate of choroidal detachment for both implants [49]. Other studies were consistent with those findings, with Schlenker et al. reporting 3 out of 181 cases of anterior chamber reformation and 4 out of 181 cases of late choroidal detachment after PreserFlo implantation [35]. Batlle and Ibarz-Barbera reported comparable results, with 13% and 11% cases of hypotony with shallow anterior chambers during the first three weeks after PreserFlo implantation [33,56].

This low incidence of hypotony might be explained by the tissue response to aqueous humor flow rate and the creation of a specific kind of filtering bleb, analyzed in a recent research using Anterior Segment Optical Coherence Tomography (AS-OCT) [56,57]. PreserFlo implantation determined the creation of fluid cavities under the Tenon from the early postoperative period to the third month, resulting in detectable horizontal and vertical expansion and a multilayered appearance of the overlaying conjunctival stroma in the majority of patients [56]. The blebs’ morphology was similar to that of trabeculectomy blebs, either in the early post-operative period, and at one-year follow-up, showing thick hypo-reflective walls. The AS-OCT morphology of the filtering blebs associated with XEN, on the other hand, has been described as low-lying, diffuse, [57] or a “filtering conjunctiva,” without a conventional bleb [58]. This may imply that the lower flow through the XEN compared with the PreserFlo probably accelerates the subconjunctival fibrotic response, increasing the number of needlings required (43–71% for XEN vs. 8.5% for PreserFlo) [35].

The safety profile of the PreserFlo MicroShunt was confirmed to be excellent in recent years, with only a few cases of sight-threatening adverse effects described in the literature. Bunod et al. described two cases of PreserFlo exposure, which required device removal, in eyes with pre-existing blepharitis and the lack of a Tenon’s flap [59]. This potentially vision-threatening complication, because of the risk of endophthalmitis, suggested that ocular surface inflammation should be detected and treated prior to device implantation [59]. The risk of endophthalmitis after PreserFlo implant surgery remains unclear, with only Brambati et al. reporting a case of ocular infection following post-operative needling procedures, managed with therapeutic pars plana vitrectomy (PPV) and antibiotics injection [54]. Few cases of choroidal detachments have been reported, generally solved with conservative approaches [60]. Micheletti et al. recently reported a case of delayed-onset hemorrhagic choroidal detachment in a patient under anti-coagulant therapy [61]. This case required surgical treatment with pars plana vitrectomy (PPV) and silicon–oil tamponade, along with PreserFlo implant removal [61]. At last, Gizzi et al. described a case of malignant glaucoma occurring 1 day after PreserFlo implant, requiring PPV in combination with clear lens extraction (CLE) and surgical irido-zonulo-hyaloidectomy, but keeping the device in place [62].

## 8. Novel Approaches

A recent retrospective study conducted by Vastardis et al. was the first to attempt Ologen collagen matrix (OCM) implantation along with PreserFlo MicroShunt surgery [63]. OCM is a porous collagen–glycosaminoglycan copolymer matrix implant that aims to modulate wound healing in connective and epithelial tissues. Positioned between the sclera surface and the conjunctiva, it acts as a spacer or barrier [64]. Although its genuine value in glaucoma surgery is still being contested [65,66], OCM is now frequently used in other ocular disorders, such as pterygium excision, where it controls conjunctival wound healing and functions as a recurrence inhibitor [67]. This study analyzed whether the OCM implant associated with PreserFlo may lower the fibrotic response in the bleb region, thus reducing revision surgery or needling treatments, when compared with PreserFlo MicroShunt implantation with MMC 0.2 mg/mL. This study showed consistent results when compared with pre-existing studies for what regards IOP reduction and safety profile [13,24,32,33,35,68]. However, no significant differences in terms of IOP reduction among the groups with or without OCM implantation were found at the 6-month follow up, with both groups having almost equal features in terms of bleb failure risk. Nevertheless, a long-term follow-up is necessary to assess whether this extra OCM implantation after PreserFlo MicroShunt surgery may offer any advantage in terms of wound modulation and bleb survival [63].

## 9. Conclusions

Severe and refractory glaucoma is still a difficult challenge for ophthalmologists due to poor adherence to pharmacologic therapy and the extensive intra- and post-operative care associated with conventional surgical procedures [1,3,4,8,69]. Trabeculectomy and large drainage tube shunts are often used to treat uncontrolled moderate-to-severe glaucoma, but often requiring intensive post-operative adjustments. PreserFlo MicroShunt surgery may be indicated early in the treatment paradigm before the optic nerve is significantly injured, and was shown to be an effective treatment option among other MIGS. PreserFlo may be used alone or in conjunction with phacoemulsification, and demonstrated sustained and predictable decreases in both mean IOP and number of glaucoma drugs even in long-term follow-ups [31,33].

Further clinical studies are being conducted in Europe, the United States, Singapore, Japan, and the Dominican Republic to confirm its effectiveness. In particular, a prospective randomized FDA clinical study is ongoing to compare the safety and effectiveness of the PreserFlo MicroShunt against trabeculectomy (NCT0188425). Another clinical study in the Netherlands (NCT03931564) will evaluate the cost-effectiveness of the PreserFlo MicroShunt versus trabeculectomy, with results to be presented in July 2022.

In conclusion, PreserFlo is set to occupy a crucial role in the glaucoma treatment armamentarium due to its unique material and design, less invasive technique for implantation, and promising effectiveness and safety profile exhibited in the aforementioned researches. Nevertheless, due to the heterogeneity of the published studies (retrospective design, no baseline data, missing statistical data, different definitions of success, changes in study protocol, inhomogeneous cohorts, and with and without cataract surgery), the current level of evidence is not yet sufficient to be able to recommend the PreserFlo device without hesitation, until the mentioned studies (FDA, Netherlands) are completed and published.

## Figures and Tables

**Table 1:** PreserFlo results in different studies, with average values reported from each research. IOP = intraocular pressure.

Study	No. of Eyes	Pre-op IOP (mmHg)	Pre-op Drugs	Follow-Up	Final IOP (mmHg)	Post-op Drugs
Riss et al. (2015) [33]	23 *	23.8	2.6	1 year	10.7	0.3
Batlle et al. (2016) [15]	23 *	23.8	2.4	3 years	10.7	0.7
Batlle et al. (2021) [34]	23 *	23.8	2.4	5 years	12.4	0.4
Beckers et al. (2017) [35]	91	24.3	2.4	1 year	13.3	0.4
Schlenker et al. (2015) [36]	164	21.4	3.4	1 year	13.3	0.5
Martinez-de-la-Casa (2021) [30]	58	21.5	2.3	1 year	14.6	0.2
Beckers et al. (2021) [32]	81	21.7	2.5	2 year	14.1	0.5

* Same cohort.

## Data Availability

The data that support the findings of this study are available from the corresponding author, M.M.C., upon reasonable request.
